# *Lactobacillus casei* culture supernatant ameliorates acute alcohol-induced liver injury by inhibiting cellular stress and promoting intestinal integrity in mice

**DOI:** 10.1371/journal.pone.0344960

**Published:** 2026-04-03

**Authors:** Xie Zhang, Zhihui Xu, Ying You, Longteng Xie, Yongsheng Jiang, Junyue Chen, Zeping Xu, Feng Xu, Deshu Zhou

**Affiliations:** 1 Department of Pharmacy, The Affiliated Lihuili Hospital of Ningbo University, Ningbo, China; 2 Department of Gastroenterology, The Second Hospital of Ninghai County, Ningbo, China; 3 College of Food science and Engineering, Jilin Agricultural University, Changchun, China; 4 Department of Gastroenterology, Xiangshan First People’s Hospital Medical and Health Group, Ningbo, China; 5 Department of Gastroenterology, The Affiliated Lihuili Hospital of Ningbo University, Ningbo, China; Jeju National University, KOREA, REPUBLIC OF

## Abstract

Alcohol-associated liver disease (ALD) is a common liver disease worldwide. Probiotics prevent alcohol-induced liver injury and improve intestinal integrity. However, the precise mechanisms behind these effects are not fully understood. In the present study, we investigated the effects of *Lactobacillus casei* (*L. casei*) AS1.2435 on ALD in a mouse model. Ten-week-old ICR mice were pretreated with *L. casei* intracellular product or *L. casei* supernatants in drinking solution for 2 weeks before gavage administration of one dose of ethanol at 6 g/kg body weight. Alcohol-induced liver injury was examined by measuring plasma alanine aminotransferase levels and histological staining of liver sections. Apoptosis in liver tissue was detected by TUNEL staining. Oxidative stress and endoplasmic reticulum stress-related proteins, including malondialdehyde, superoxide dismutase, glutathione peroxidase, C/Ebp-homologous protein, glucose-regulated protein 78, protein disulfide isomerase, and X box-binding protein-1 in liver tissues were measured using commercial kits and immunohistochemical analysis. Intestinal tissue was examined by histological staining, and the expression of tight junction mRNA and proteins were analyzed by RT-PCR and immunofluorescence. *L. casei* supernatants supplementation significantly reduced alcohol-induced liver fat accumulation, inflammatory responses, intestinal mucosa injury, and improved liver function. *L. casei* supernatants prevented alcohol-induced increases in apoptosis, oxidative damage, and endoplasmic reticulum stress in mouse livers. *L. casei* supernatants pretreatment significantly increased alcohol-reduced mRNA expression of zonula occludens-1, claudin-1, intestine trefoil factor, P-glycoprotein, and cathelin-related antimicrobial peptide. Consistently, the increased protein levels of ZO-1, occludin, and claudin-1 were confirmed by immunofluorescence. However, no effects were observed in the *L. casei* intracellular product pretreatment groups. *L. casei* supernatants’ extracellular product can alleviate ALD and intestinal injury by inhibiting cellular stress and promoting intestinal integrity.

## Introduction

Alcohol-associated liver disease (ALD) is a syndrome that includes a range of findings from simple liver steatosis to steatohepatitis, followed by progressive alcoholic liver fibrosis and cirrhosis, chronic hepatitis, and finally, extensive hepatocyte necrosis or liver cancer [1,2]. The pathogenesis of ALD is unclear; however, mechanisms may include oxidative stress, inflammatory responses, endoplasmic reticulum (ER) stress, or apoptosis [3–5]. Ethanol exposure affects levels of hepatic antioxidants such as glutathione (GSH), superoxide dismutase (SOD), and glutathione peroxidase (GSH-Px) activity [3]. Oxidative damage to macromolecules in hepatocytes altered mitochondrial function. Injury to hepatocyte mitochondria can interfere with the oxidation of beta fatty acids, reduce the production of adenosine triphosphate, and lead to the deposition of triglyceride (TG) in the liver. In addition, injury can induce apoptosis by regulating mitochondrial apoptosis signaling molecules such as Bcl-2, Bax, and caspases [5]. Excessive alcohol intake can induce ER stress by inducing acid phospholipase synthesis, interfering with calcium homeostasis, and aggravating liver inflammation and oxidative damage [4]. Hepatocyte apoptosis is a pathogenic event in ALD and may be associated with unresolved ER stress. In ALD, misfolded proteins accumulate in the ER, which is sensed by glucose-regulated protein 78 (GRP78) leading to the activation of related protein genes, such as C/EBP-homologous protein (CHOP) [6].

Studies showed that alcohol increases intestinal permeability, releases lipopolysaccharide (LPS) from gram-negative bacteria, activates immune responses [7], and damages the gastrointestinal barrier, resulting in reduced expression of intestinal tight junction proteins, including claudins, zonula occludens-1 (ZO-1), and occludin, causing intestinal epithelial cells to create highly permeable paracellular spaces [8]. With the increase of the permeability of the intestinal mucosal barrier, endotoxin and pathogens can pass through the intestinal wall, enter the portal vein system, activate Kupffer cells (a type of macrophages) in the liver, and release inflammatory factors such as tumor necrosis factor-α (TNF-α) and interleukins (including IL-1 β and IL-6) [9,10], and further lead to the intestinal mucosa and liver damage.

Probiotics are live microorganisms that confer benefits to hosts when consumed in adequate amounts (FAO/WHO) [11,12]; they are widely used in the food and medical industries [13,14]. A growing body of evidence suggests that probiotics protect against ALD by regulating systemic immune function, adjusting the intestinal mucosal barrier, and balancing gut flora and nutrition [15]. *Lactobacillus* (LAB) is the most widely used probiotic in the pharmaceutical and food fields because of its antineoplastic and immunoregulatory functions. In a study of ten patients with alcoholic cirrhosis treated with a mixture of probiotic strains*,* including *Lactobacillus,* Loguercio found that alanine aminotransferase (ALT) and TNF-α levels decreased, suggesting that *Lactobacillus* may treat liver disease [16]. Probiotics such as *Bifidobacterium* or *Lactobacillus,* improved ALD by restoring intestinal flora and regulating liver enzymes in animal and clinical studies [17]. *Lactobacillus casei* (*L. casei*) is among the most well-studied probiotics. Some strains have a protective effect on alcoholic liver injury. An animal study reported therapeutic effects after supplementing *L. casei* with alcoholic cirrhosis [18]. A clinical study showed that supplementation with *L. casei* improved lipid metabolism and modulated intestinal dysbiosis in patients with alcoholic liver injury [19]. Another study showed that Cell-free supernatant was as effective as intact cells [20,21]. However, no studies focused on the protective effect of *L. casei* on acute alcoholic liver injury. Therefore, the present study aimed to investigate the protective effects and mechanisms of *L. casei* on acute alcoholic liver injury using intracellular and extracellular products.

## Materials and methods

### Preparation of *Lactobacillus casei products*

*Lactobacillus casei* AS1.2435 products were purchased from Shanghai Xuanya Biotechnology Co, Ltd (Shanghai, China). *L. casei* was activated and passaged three times in MRS medium, inoculated in 100 mL liquid medium (3% inoculation in culture medium), cultured at 37 ℃, 5% CO_2_ for 24 hours, and centrifuged at 5,000 g for 10 min at 4 ℃. This procedure yielded *L. casei* supernatants (LC-cs) from the culture at 10^9^ colony-forming units/ml bacterial cells. The supernatant was filtered through 0.22-μm filters to obtain extracellular fluid as the extracellular product. The precipitate was subjected to ultrasonication (ultrasonic work rate 300–350 W, working for 9.9 s, intermittent 9.9 s, 152 cycles) to lyse cell walls on ice and obtain *L. casei* intracellular components (LC-ic). After LC-ic or LC-cs incorporation (diluent of 1:20), the solution tasted somewhat sour and sweet. Daily consumption was about 8–9 ml (10^9^ CFU) per mouse.

### Animal experiment

Forty male ICR mice, 10 weeks old, were obtained from the Animal Center of the Chinese Academy of Sciences (Shanghai, China) and housed at the Wenzhou Medical University animal facility. Healthy mice were randomly divided into four groups (ten mice each): Control, ethanol (EtOH), LC-ic + EtOH, and LC-cs + EtOH groups. The acute alcohol-induced liver injury model was established as previously described [22–24]. Briefly, mice in the LC-ic + EtOH and LC-cs + EtOH groups were pretreated with LC-ic or LC-cs in a drinking solution for two weeks before a single dose of ethanol (6 g/kg body weight) was administered by gavage after overnight fasting, with access to a drinking solution containing LC-ic or LC-cs; this condition continued for 6 hours. The control mice were administered the same volume of saline by gavage.

Six hours after alcohol or saline administration, all mice were deeply anesthetized via an intraperitoneal injection of sodium pentobarbital (50 mg/kg). Every effort was made to minimize suffering throughout the procedure. Blood was collected via cardiac puncture. Subsequently, euthanasia was performed by slow-fill carbon dioxide (CO₂) inhalation, followed by cervical dislocation as a secondary method to ensure death. The liver and duodenum were cryopreserved or fixed in paraformaldehyde. All mice were treated according to protocols approved by the Institutional Animal Care and Use Committee of Wenzhou Medical University.

### Biochemical analysis

Plasma was obtained by centrifuging the blood at 2,000 *g* for 30 min at 4 °C. Plasma levels of ALT and LPS were measured using diagnostic ALT ELISA kits (Nanjing Jiancheng Bioengineering Institute, China) [25] and Limulus Amebocyte Lysate test kits (Lonza, Walkersville, MD) [26] according to the manufacturer’s instructions.

### Hepatic Triglyceride assay

Liver tissue was homogenized in 1 ml of 50 mM NaCl and centrifuged at 1,800 g for 20 min at 20℃for the liver triglyceride(TG) assay using a TG Kit (Thermo Fisher Scientific Inc.) [23].

### Liver TNF-α and IL-6 assay

Liver tissue was homogenized in RIPA buffer (50mM Tris-HCl, ph 7.4, 150mM NaCl, 2mM EDTA, 4mM Na_3_VO_4_, 40mM NaF, 1% Triton X-100, 1 mM phenylmethylsulfonyl fluoride, 1% protease inhibitor cocktail). TNF-α and IL-6 were measured using assay kits (Thermo Scientific, MD,USA), according to the manufacturer’s instructions.

### Measurements of antioxidant markers in liver tissue

The liver tissues were homogenized in cold Tris-HCl. The homogenates were centrifuged (2500 *g* for 10 min at 4 °C), and the supernatants were collected. Levels of malondialdehyde (MDA), superoxide dismutase (SOD), total antioxidant capacity (T-AOC), and glutathione peroxidase (GSH-Px) in liver tissues were determined using commercial kits (Beyotime Biotechnology Corporation, Shanghai, China) [25,27].

### Histological analysis and immunohistochemistry

Liver and intestinal tissue (duodenum) were fixed in 10% formalin solution, embedded in paraffin, cut into 4-μm sections, and stained with hematoxylin-eosin. For immunohistochemistry, after deparaffinization and rehydration, the transverse paraffin sections were also incubated in 3% H_2_O_2_. Subsequently, the sections were incubated at 4 ℃ overnight with the following primary antibodies: C/Ebp-homologous protein (CHOP; 1:200) [28], glucose-regulated protein 78 (GRP78; 1:200) [29], protein disulfide isomerase (PDI; 1:300) [30] and X box-binding protein-1 (XBP-1, 1:200) [31]. After triple washing in PBS, the sections were incubated with secondary antibodies for 2 h at 37 °C. All images were captured on a Nikon ECLIPSE Ti microscope.

### Liver oil red O staining

Frozen liver tissue sections were processed at room temperature for 30 min for staining with oil red O and then studied by light microscopy.

### Terminal deoxynucleotidyl transferase deoxyuridine Triphosphate Nick End Labeling Assay

Paraffinized samples (4-mm) were removed from the sections with xylene, rehydrated in graded alcohol series, and subjected to antigen retrieval in 0.01 M citrate buffer (pH 6.0) by microwaving. Sections were then incubated with 20 mg/ml proteinase K for 15 minutes. The sections were incubated with terminal deoxynucleotidyl transferase enzyme, fluorescein-deoxyuridine-triphosphate, and TUNEL extract (1:24:25) at 37 °C for 1 hour (Beyotime). Then, the sections were viewed under the Nikon ECLIPSE Ti microscope. For TUNEL staining, kernels stained in blue with Harris hematoxylin were assessed as normal, and cells demonstrating bright green fluorescence nuclear staining were established as apoptotic. TUNEL-positive cells were counted. The results were quantified and photographed using a Nikon ECLIPSE Ti microscope.

### Real-time RT-PCR assay

The mRNA levels were detected using real-time RT-PCR. Total RNA was briefly isolated using TRIzol reagent according to the manufacturer’s instructions (Invitrogen, Carlsbad, CA) and reverse-transcribed with the GenAmp-RNA polymerase chain kit (Applied Biosystems, Foster City, CA). The sequences of the primers were as follows: Zonula occludens-1 (ZO-1), forward, 5’-TGGGAACAGCACACAGTGAC-3’, reverse, 5’-GC TGGCCCTCCTTTTAACAC-3’; occludin, forward, 5’-ACCCGAAGAAAGATGGATCG- 3’, reverse, 5’-CATAGTCAGATGGGGGTGGA-3’; claudin-1, forward, 5’-CGGGCAGA TACAGTGCAAAG-3’, reverse, 5’-ACTTCATGCCAATGGTGGAC-3’; Intestinal trefoil factor (ITF), forward, 5’- TGGGATAGCTGCAGATTACG-3’, reverse, 5’-GCCACAGTCCACTCTGACAT-3’; P-glycoprotein (P-gp), forward, 5’-GTGGGGGACAGAAACAGAGA-3’, reverse, 5’-GAACGGTAGACA AGCGATGAG-3; cathelin-related antimicrobial peptide (CRAMP), forward, 5’-CAGCCCTTTCGGTTCAAGAA-3’, reverse, 5’- CCCACCTTTGCGGAGAAGRT-3’; β-actin, forward, 5’-GGCTGTATTCCCCTCCATC G-3’, reverse, 5’-CCAGTTGGTAACAATGCCATGT-3’. The relative amounts of target transcripts were calculated from the repeated samples after normalizing the data against the housekeeping gene, β-actin. The expression of relative mRNA was calculated using the ^ΔΔ^Ct method.

### Immunofluorescence staining

Immunofluorescence staining for ZO-1, occludin, and claudin-1 on duodenum sections was performed as previously described [24] using specific primary antibodies against ZO-1 (1:200, sc-33725), occludin (1:200, sc-133256), and claudin-1 (1:200, sc-166338).

### Statistical analysis

The data were expressed as the mean ± standard error of the mean. Statistical calculations were performed using GraphPad Prism 5 (GraphPad Software, Inc., San Diego, USA). The data were analyzed using analysis of variance and Newman–Keuls multiple-comparison test. *P* < 0.05 was considered statistically significant.

## Results

### LC-cs decreased alcohol-induced liver injury in mice

**Fig 1:**
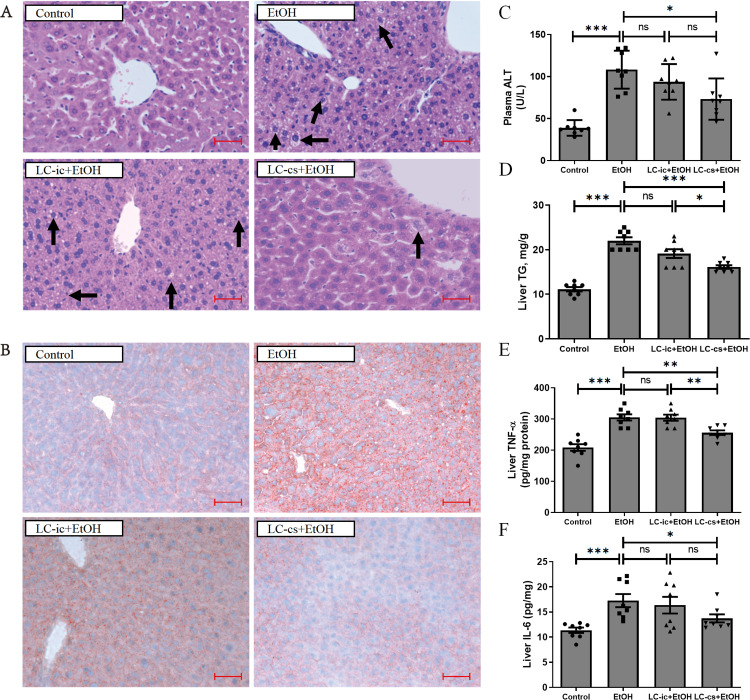
Effect of *Lactobacillus casei* (*L. casei*) pretreatment on acute alcohol-induced liver injury. **(A)** Hematoxylin and eosin (H&E) staining of livers from Control, EtOH, LC-ic + EtOH and LC-cs + EtOH mice (40 x: scale bars = 25 µm). **(B)** Oil Red O staining of livers from Control, EtOH, LC-ic + EtOH and LC-cs + EtOH mice (20 x: scale bars = 50 µm). **(C)** Plasma alanine aminotransaminase (ALT) levels. **(D)** Liver triglyceride (TG) levels. **(E)** Liver TNF-α levels. **(F)** Liver IL-6 levels. Data are expressed as mean ± SEM. n = 8, *p < 0.05, **p < 0.01 and ***p < 0.001.

As described, mouse livers were obtained and stained with hematoxylin and eosin (H&E) (Fig 1A) and Oil Red O (Fig 1B). Staining sections showed that treatment with *L. casei* extracellular product markedly reduced liver lipid droplet accumulation, which increased after alcohol exposure (Fig 1A, 1B). We found that acute alcohol exposure dramatically elevated liver TG levels compared to the control group. This effect was reversed with *L. casei* extracellular product (Fig. 1D). Nevertheless, the intracellular products of *L. casei* had no significant effect on alcohol-induced liver injury or hepatic fat accumulation compared with the control group (Fig 1D).

When liver cells are damaged, ALT is released into the circulation in large quantities, reflecting the degree of liver injury [32]. We found that the LC-cs + EtOH group showed lower increases in ALT levels after alcohol administration; however, this did not occur in the LC-ic + EtOH group (Fig 1C). Levels of inflammatory factors in the liver, including TNF-α and IL-6, were also analyzed. After alcohol treatment, TNF-α and IL-6 were significantly higher, suggesting that inflammation occurred in the liver (Fig 1E and 1F). This change was blunted in the LC-cs + EtOH group, while there was no significant effect in the LC-ic + EtOH group. These results suggest that LC-cs alleviate alcohol-induced liver damage.

### LC-cs inhibited alcohol-induced hepatocyte apoptosis in mice

**Fig 2:**
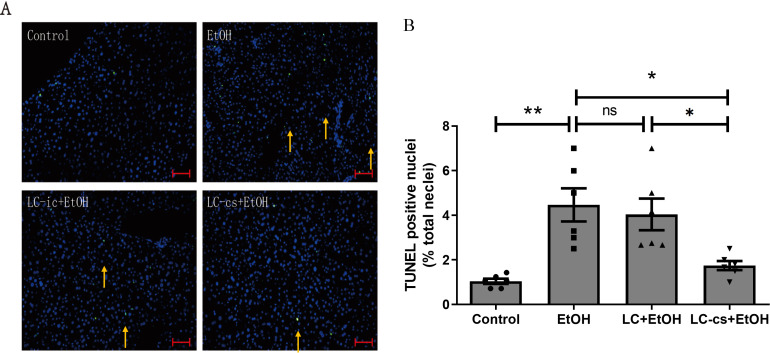
Effect of *Lactobacillus casei* (*L. casei*) pretreatment on alcohol-induced hepatocyte apoptosis. **(A)** TUNEL-positive nuclear staining of liver from Control, EtOH, LC-ic + EtOH and LC-cs + EtOH mice (20 x: scale bars = 100 µm). **(B)** Quantification of the relative number of TUNEL-positive cells. Data are expressed as mean ± SEM. N = 6, *p < 0.05 and **p < 0.01.

To investigate the effect of *L. casei* on alcoholic liver injury, we performed TUNEL staining for apoptosis detection in liver tissue sections. Compared with the control group, the number of apoptosis-positive cells (bright green fluorescence) was significantly higher after alcohol treatment, and this phenomenon was inhibited after pretreatment with *L. casei* extracellular product. In contrast, this phenomenon was not seen with the intracellular product (Fig 2A, 2B). These results suggest that LC-cs may alleviate alcohol-induced liver tissue damage by reducing apoptosis.

### LC-cs protected against alcohol-induced hepatic oxidative stress in mice

**Fig 3:**
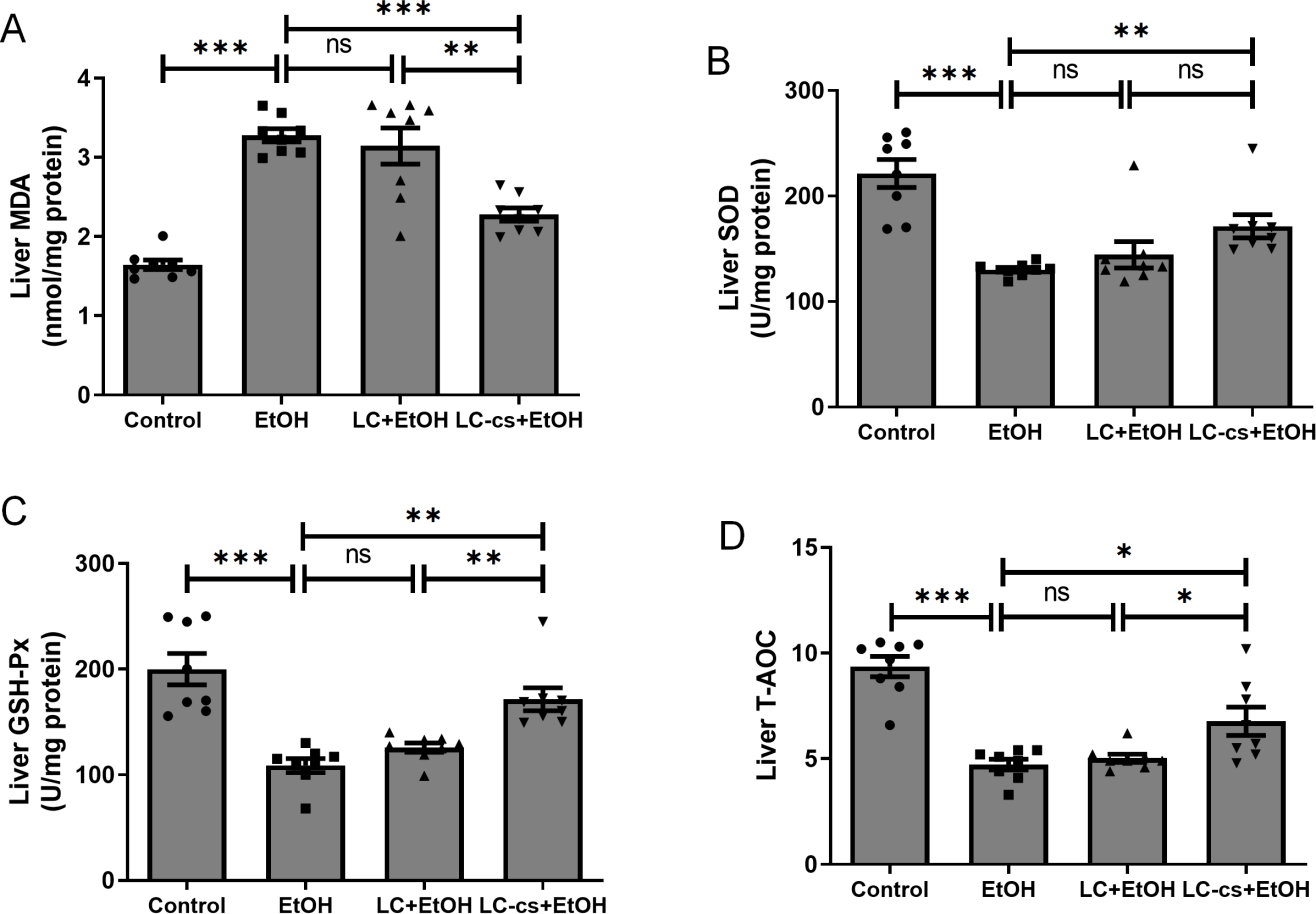
Effect of *Lactobacillus casei* (*L. casei*) pretreatment on alcohol-induced liver oxidative stress. **(A)** Liver malondialdehyde (MDA) levels. **(B)** Liver superoxide dismutase (SOD) levels. **(C)** Liver glutathione peroxidase (GSH-PX) levels. **(D)** Liver total antioxidant capacity (T-AOC) levels. Data are expressed as mean ± SEM. n = 8, *p < 0.05, **p < 0.01 and ***p < 0.001.

Alcohol intake can lead to increased levels of reactive oxygen species. As shown in Fig 3, compared with the control group, alcohol exposure significantly increased MDA levels and significantly decreased SOD, GSH-Px, and T-AOC, suggesting alcohol-induced hepatic oxidative damage. In contrast, treatment with LC-cs relieved this damage by increasing hepatic activities of SOD, T-AOC, and GSH-Px and decreasing MDA levels, while *L. casei* intracellular product did not have this effect. These results suggest that *L. casei* extracellular has potent antioxidant effects and significantly reduces alcohol-induced hepatic oxidative stress.

### LC-cs reduced alcohol-induced ER stress in the liver

**Fig 4:**
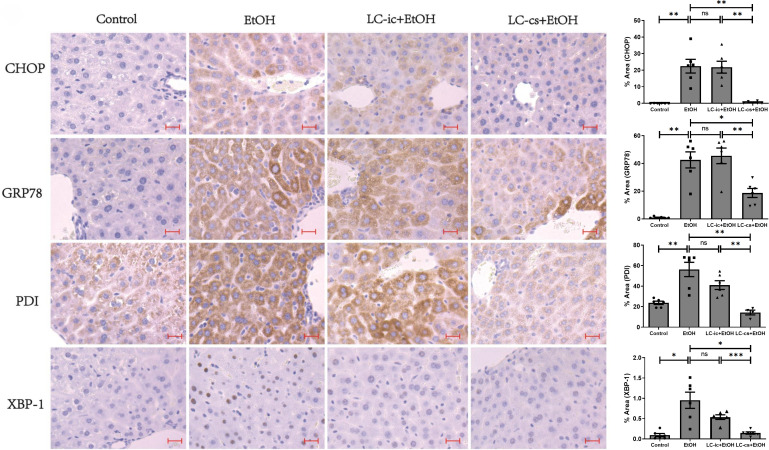
Effect of *Lactobacillus casei* (*L. casei*) pretreatment on alcohol-induced liver endoplasmic reticulum stress. Immunohistochemistry staining of C/Ebp-homologous protein (CHOP), glucose-regulated protein 78 (GRP78), Protein disulfide isomerase (PDI) and X box-binding protein-1 (XBP-1) positive area of liver from Control, EtOH, LC-ic + EtOH and LC-cs + EtOH mice (40 x: scale bars = 50 µm, n = 6).

ER is abundant in liver cells, and the destruction of ER homeostasis is a major cause of liver apoptosis. To evaluate the role of ER stress in alcohol-induced hepatotoxicity, immunohistochemical analysis was used to measure levels of the ER stress-associated proteins CHOP, GRP78, PDI, and XBP1 (Fig 4). All were all upregulated at 6 h after alcohol gavage and were significantly downregulated by LC-cs pretreatment. Compared with the control group, *L. casei* intracellular product did not significantly reduce ER stress-related protein levels. These results suggest that LC-cs prevent alcohol-induced hepatotoxicity by inhibiting ER stress.

### LC-cs ameliorated alcohol-induced intestinal barrier injury and endotoxemia leakage

**Fig 5:**
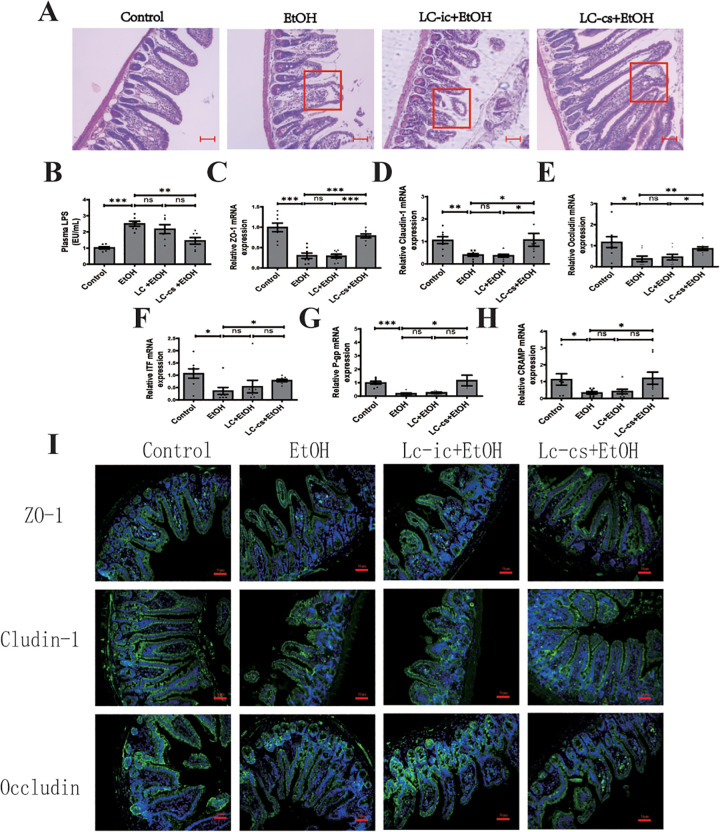
Effect of *Lactobacillus casei* (*L. casei*) pretreatment on alcohol-induced intestinal barrier injury and endotoxemia. **(A)** Hematoxylin and eosin (H&E) staining of intestine from Control, EtOH, LC-ic + EtOH, and LC-cs + EtOH mice (40 x: scale bars = 25 µm). **(B)** Plasma LPS levels. **(C-H)** Relative intestine mRNA expression of intestinal trefoil factor (ITF), P-gp, cathelin-related antimicrobial peptide (CRAMP), zonula occludens-1 (ZO-1), occludin and claudin-1 levels was detected. **(I)** Representative immunofluorescence staining of the key tight junction proteins ZO-1, occludin, and claudin-1 in the duodenum (200 x: scale bars = 50 µm). Data are expressed as mean ± SEM. n = 6-8, *p < 0.05, **p < 0.01 and ***p < 0.001.

To analyze the effects of the *L. casei* product on intestinal barrier function, the intestinal tissues were obtained and stained with H&E. As shown in Fig 5A, the structure of duodenum villi is destroyed, the folds are reduced, the structure of the central lacteal is destroyed, and the shape disappears. The striated margin is discontinuous in the alcohol treatment group. The *L. casei* extracellular product treatment inhibited these effects, while the intracellular product did not.

Gut injury and intestinal barrier destruction are two leading causes of elevated plasma LPS levels [33]. Our results also showed that *L. casei* extracellular product attenuated alcohol-induced increases in LPS, while this effect was not significant in the LC-ic + EtOH group (Fig 5B).

Tight junctions (TJs) play an essential role in the intestinal epithelial barrier. Real-time qPCR results revealed that compared with the normal group, alcohol treatment reduced mRNA expression of ZO-1, occludin, and claudin-1 in ileum epithelium, *L. casei* extracellular product pretreatment reversed these reductions, and *L. casei* intracellular product did not have this effect (*P >* 0.05, Fig 5C-5E). Consistently, immunofluorescence staining of the duodenum demonstrated that alcohol exposure disrupted the distribution and continuity of ZO-1, occludin, and claudin-1 proteins at the epithelial barrier, which was effectively preserved by LC-cs pretreatment (Fig 5I).

ITF, P-gp, and CRAMP are vital ileal barrier protective proteins and play essential roles in maintaining intestinal epithelial integrity [34]. Similar to TJs, ITF, P-gp, and CRAMP mRNA levels were significantly reduced by alcohol exposure and normalized by *L. casei* extracellular product pretreatment but not by intracellular product pretreatment (Fig 5F-5H). These results suggest that LC-cs pretreatment protects against alcohol-induced intestinal barrier compromise.

## Discussion

We investigated the effects of supernatants obtained from *L. casei* on alcohol-induced hepatic injury and intestinal epithelial cell permeability. Numerous reports also showed that alcohol-induced oxidative stress, ER stress, and inflammation contributed to liver injury and intestinal damage [33,35,36]. The present study demonstrated that acute alcohol administration caused significant liver fat accumulation, inflammatory responses, and intestinal mucosa injury, consistent with previous studies [22,23]. Treatment with LC-cs reversed these effects. In addition, *L. casei* culture supernatants alleviated alcohol-induced liver injury by inhibiting apoptosis, inflammatory cytokine production, oxidative damage, and ER stress in mouse livers. This alcohol-induced reduction in the mRNA expression of tight junction- and barrier protective proteins was also restored by LC-cs pretreatment, as confirmed at the protein level by immunofluorescence staining showing preserved distribution of ZO-1, occludin, and claudin-1.

Currently, abstinence is the best treatment for all stages of ALD, but it may be difficult for heavy drinkers. Meanwhile, several lines of evidence suggest that probiotics protect against ALD [15,17,35,37], thereby increasing interest in developing probiotic treatments. Similar to these studies, *Lactobacillus* alleviated alcohol-induced increases in ALT and AST levels and inhibited oxidative stress and inflammatory responses in the liver, suggesting that *Lactobacillus* prevents alcohol-induced liver injury [35]. The protective effects of probiotics against ALD are complicated. Wang et al. found that acute alcohol exposure increased intestinal permeability and induced liver injury; however, *Lactobacillus rhamnosus* GG (LGG)-supernatant treatment abrogated these effects by restoring the expression of adhesion proteins such as claudin-1 and ITF [23], suggesting that these supernatants protect against acute/chronic alcoholic liver injury. Alcohol alters the proportions and diversity of bacteria and other microorganisms in the intestinal tract and disrupts intestinal homeostasis. Transplantation of fecal flora in the intestinal tract of alcohol-resistant mice restored the proportion and diversity of intestinal microorganisms in alcohol-sensitive mice and prevented ALD [38]. Clinical studies showed that probiotic treatment enriched intestinal flora and reduced gram-negative bacteria, thereby decreasing LPS production and preserving intestinal homeostasis [17,39].

Alcohol consumption leads to intestinal and liver damage in humans and laboratory animals. Alcohol consumption can cause intestinal and liver damage in humans and laboratory animals. Tian found that treatment with *L. plantarum* CCFM1107 prevented ALD and repaired injured intestines [40]. Similarly, a recent study also showed that probiotic compounds composed of bifidobacterium and Lactobacillus reversed intestinal flora imbalance caused by acute alcohol intake, maintained intestinal barrier integrity, reduced inflammation by inhibiting the TLR4/ NF-κb signaling pathway, and reduced liver oxidative stress levels. As these results, the present study identified the protective effects of *L. casei* supernatant against alcohol-induced intestinal and liver damage. Treatment with L. casei extracellular product markedly reduced liver lipid droplet accumulation, which increased after alcohol exposure. Levels of inflammatory factors in the liver, including TNF-α and IL-6, were significantly reduced after LC-cs intervention.

Another factor contributing to alcohol is liver metabolism. Long-term ethanol intake stimulates hepatocytes to produce large amounts of reactive oxygen species with strong oxidative properties, including elevating serum ALT and AST levels [27] and producing lipid peroxides, such as MDA and 4-hydroxynonenal, which can lead to hepatocyte damage. Oxidative stress further promotes ER stress in the liver. In mice with chronic alcoholic hepatitis, pretreatment with *Lactobacillus plantarum* CMU995 improved liver GSH and SOD levels, reduced intrahepatic GSH S-transferase activity and reduced lipid peroxidation [41]. LGG supplementation inhibited ER stress and the IRE1α/XBP-1 signaling pathway, thereby modulating the intestinal microbiota [42]. The present study found that LC-cs restored alcohol-induced liver damage by suppressing oxidative damage and ER stress (Figs 3 and 4). Alcohol exposure significantly increased MDA levels and significantly decreased the activities of SOD, GSH-Px, and T-AOC. In contrast, treatment with LC-cs relieved this damage by increasing hepatic activities of SOD, T-AOC, and GSH-Px and decreasing MDA levels. In addition, the ER stress-associated proteins CHOP, GRP78, PDI, and XBP-1 were upregulated significantly at 6 h after alcohol gavage and significantly downregulated by LC-cs pretreatment.

Some strains of *Lactobacillus* have been evaluated in patients with nonalcoholic fatty liver disease and in rodent models. Recently, some studies focused on *L.* casei. Aktas et al. reported that *L. casei* altered the composition of the intestinal microbiota and modulated the host immune response [43]. Xuelong Li et al. investigated clinical ALD patients and showed that supplementation with *L. casei* improved lipid metabolism and regulated intestinal flora disturbance [19]. Other studies found that *L. casei* helps maintain intestinal flora balance, supporting intestinal barrier integrity and reducing liver inflammation and oxidative stress in rats. We used an acute alcohol mouse model to reproduce the effects of *L. casei* on these aspects and found that LC-cs significantly reduced alcohol-induced liver and intestinal injury. Some active ingredients must regulate intestinal flora, enhance immune responses, protect against intestinal barrier dysfunction, and combat hepatic steatosis; however, it is unclear which specific active ingredients need further analysis..

Previous studies showed that most intervention methods used were whole bacteria or supernatant. Koga et al. studied ALD patients and found that *L. casei* intervention regulated intestinal flora homeostasis [44]. Wang et al. reported that *Lactobacillus* rhamnosus supernatant maintained the integrity of the intestinal barrier and attenuated endotoxemia-driven liver injury [23]. Sharma C et al. observed a significant reduction in the pathogen-inhibitory abilities of the probiotic *Lactobacillus reuteri* in heat-inactivated forms compared to viable forms [45]. However, other evidence showed that heat-killed lactic acid bacteria cells could also reduce inflammation and oxidative stress in alcoholic liver injury. Studies have found that live lactic acid bacteria can protect intestinal cells from pathogenic bacteria such as Salmonella and Escherichia coli according to their intestinal adhesion [16,46,47]. In contrast, heat-killed strains of *Lactobacillus acidophilus* protect against the invasion of pathogenic bacteria by activating the intestinal immune system, but the specific components have not been elucidated.

*L. casei* protects against chronic alcoholic liver injury [19]. In the present study, we used *L. casei* intracellular and extracellular products to study the protective effect of *Lactobacillus casei* on acute alcoholic liver injury. Several studies showed that *Lactobacillus* live, supernatant, and heat-killed *Lactobacillus* may mediate their beneficial effects; however, the specific active ingredients remain unclear. In this study, LC-cs yielded the same result as a previous study [23], whereas intracellular products were not significant in all aspects, suggesting that the effect of *L. casei* in regulating ALD through intestinal-liver action is primarily due to LC-cs. The reason the intracellular components of this experiment differ from those in previous experiments might be that heat-killed lactic acid bacteria retain their cellular structure, whereas the cellular structure in our experiment has been destroyed. Previous studies found that dead bacterial cells (with no intracellular components but intact cellular structure) may limit the adhesion of pathogens to epithelial surfaces [47–49], suggesting that the cellular structure itself plays a role in regulating intestinal microbiota homeostasis. Several studies showed that probiotic stimulation of growth factor secretion might mediate their beneficial effects; in fact, some factors in probiotic culture supernatants might be active ingredients, including conjugated linoleic acid, polyamines, peptides, and polyphosphates [23,35,50,51]. Indeed, a recent study showed that LC-cs were effective as intact cells, and LC-cs contain multiple compounds with antibacterial activity, including organic acids, such as lactic acid, hydrogen peroxide, and bacteriocins, which play an essential role in protecting against alcoholic liver injury [51]. Therefore, identifying the full spectrum of active ingredients in LC-cs culture supernatant in response to alcohol exposure warrants further attention.

Despite the compelling protective effects of LC-cs demonstrated in this study, several limitations should be acknowledged. While our work comprehensively delineates the protective phenotype of LC-cs against acute alcohol-induced liver and intestinal injury, it is primarily observational. A key limitation is the lack of functional validation using specific inhibitors or genetically modified animals to pinpoint the exact upstream signaling pathways (e.g., Nrf2, TLR4, or specific ER stress sensors) responsible for the observed reductions in cellular stress and improvements in intestinal integrity. For instance, recent studies have employed such approaches to definitively establish the roles of the Nrf2/HO-1 antioxidant pathway [52–54], TLR4/NF-κB inflammatory pathway [52,54,55], and ER stress sensors (e.g., IRE1α, PERK, XBP-1) [53,56,57] in mediating protection against intestinal injury, which could serve as a reference for future mechanistic exploration of our findings. Furthermore, the specific active ingredients within the supernatant responsible for these protective effects remain to be fully elucidated. Future research employing metabolomics [58], supernatant fractionation, and in vitro models will be essential to identify these components, which may include organic acids, bacteriocins, or other secreted metabolites. In this regard, utilizing in vitro systems, such as hepatocyte and intestinal organoid cultures, will be invaluable for dissecting the direct cellular targets and signaling pathways modulated by LC-cs, independent of the complex in vivo microenvironment. Therefore, while our findings establish a solid foundation for the therapeutic potential of LC-cs, they also chart a clear course for future mechanistic investigations to fully elucidate their molecular targets and active constituents.

We demonstrated that *L. casei* extracellular products were superior to intracellular products in reducing alcohol-induced damage to the liver and intestine. Acute alcohol administration increased fat accumulation, activated inflammatory liver responses, and damaged intestinal barrier function, while the *L. casei* extracellular product reversed all these effects. These effects may have been mediated by inhibition of hepatic oxidative stress, ER stress, hepatocyte apoptosis, and intestinal injury repair. Our findings provide a theoretical basis for the development of functional foods.

## Supporting information

S1 Fig Body weights of mice in different groups before and after the 2-week pretreatment with *Lactobacillus casei* (*L. casei*) pretreatment. Datas are expressed as mean ± SEM, n = 10.
(TIF)
