# Supplementary figures and images for: Lactobacillus casei culture supernatant ameliorates acute alcohol-induced liver injury by inhibiting cellular stress and promoting intestinal integrity in mice
Source: PLoS One. 2026 Apr 3;21(4):e0344960. doi: 10.1371/journal.pone.0344960 (PMC13048434; doi:10.1371/journal.pone.0344960)

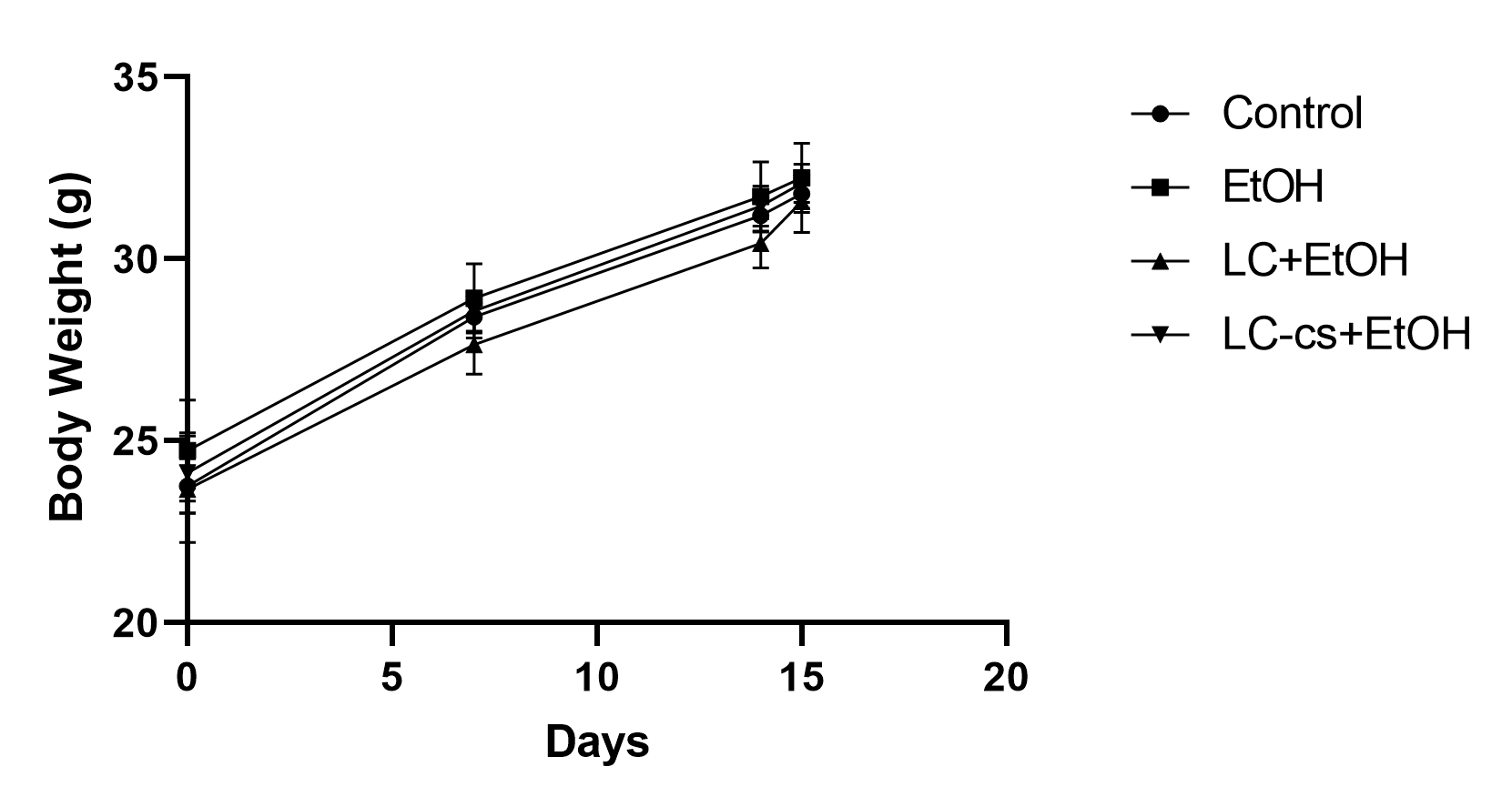

Supplement: S1 Fig — Datas are expressed as mean ± SEM, n = 10. (TIF) [file pone.0344960.s001.tif]
